# Ovine Paratuberculosis: A Seroprevalence Study in Dairy Flocks Reared in the Marche Region, Italy

**DOI:** 10.4061/2011/782875

**Published:** 2011-08-24

**Authors:** Attili Anna Rita, Ngu Ngwa Victor, Preziuso Silvia, Pacifici Luciana, Domesi Anastasia, Cuteri Vincenzo

**Affiliations:** ^1^School of Veterinary Medical Sciences, University of Camerino, via Circonvallazione 93-95, 62024 Matelica, Italy; ^2^Regione Marche, Azienda Sanitaria Unica Regionale 10, Servizio Veterinario Sanità Animale, via Muralto, 62032 Camerino, Italy

## Abstract

In order to fulfil the seroprevalence gap on *Mycobacterium avium* subsp. *paratuberculosis* infection in ovine dairy farms of Marche region (central Italy), a stratified study was carried out on 2086 adult female sheep randomly chosen from 38 herds selected in Ancona and Macerata provinces. 73.7% flocks resulted infected by a commercial ELISA test (Pourquier, France), with a mean seroprevalence of 6.29% of sampled sheep in both provinces. A higher number of *MAP* seropositive ewes was recorded in the large herds' consistence than in the small and medium herds' consistence (*P* = 0.0269), and a greater percentage of infected sheep was obtained among female at early/late than in peak lactation stage (*P* = 0.0237). *MAP* infection was confirmed in 12.6% of infected farms by faecal culture. The true sheep-level seroprevalence was 15.1% ± 7.3%.

## 1. Introduction

Paratuberculosis, known as Johne's disease (JD), is a prevalent and economically important chronic, nontreatable inflammatory bowel disease of domestic and wild ruminants as well as other mammals worldwide, including nonhuman primates [1–4]. It is on the list of “multiple species diseases” notifiable to the World Organization for Animal Health as a ruminant disease of concern [5]. The ovine paratuberculosis is an object of worry since its insidious evolution and the severity of the illness are always accompanied to a too late clinical diagnosis. It represents a sanitary and zootechnical problem of remarkable proportions because of its incidence, the lack of a valid therapeutic and preventive strategy, and the economic losses due to clinical and subclinical disease [2, 6, 7].

*Mycobacterium avium* subspecies *paratuberculosis* (*MAP*) has also been suggested as an aetiological agent of Crohn's disease, a chronic, granulomatous infection of the human intestine [8–10]. The bacterium has been isolated from a high percentage of Crohn's patients [11–13] although the zoonotic potential of the organism remains controversial [8, 14]. More recently, its involvement was hypothesized for the onset of human Type I diabetes [15, 16]. People are exposed to *MAP *by direct contact with infected material and in retail milk supplies [17]. Ruminant milk has been described as a potential source through which human beings could be infected [18, 19]. Viable *MAP* has been isolated from milk and colostrum of clinically and subclinically infected cows [20], and infection of the mammary gland has been documented in small ruminants [21]. *MAP *has been demonstrated by PCR in goats' milk from bulk tanks in farms in the UK [22], the bacterium can survive pasteurisation, and cheese production processes have been shown to have little effect on the viability of *MAP* [18, 23]. Furthermore, viable bacteria have been demonstrated in hard and semi-hard cheese 120 days after production [24].

Diagnosis of subclinical infection with *MAP* in ruminant species remains one of the greatest challenges to JD control, both at the individual animal [4, 25] and herd level [26].

High shedders of *MAP* and animal with clinical signs of paratuberculosis are responsible for the greater part of the contamination of their environment, the economic damage in infected herds, and the presence of bacteria in milk [27]. To monitor the progression of a control programme, the herds need to be tested. Serology is the most practical method used for this purpose. The enzyme-linked immunosorbent assay (ELISA) is a suitable diagnostic tool to detect serum antibodies against *MAP* on a large scale, because it is possible to test large numbers of samples with a high reproducibility [25, 28]. In general, commercially available ELISA-kits for paratuberculosis have a low sensitivity [29–31]. Nevertheless, the assays have a reasonable good sensitivity to identify heavy shedders for culling in a preclinical stage [32, 33], reduce the pressure of infection in infected herds, and thus enhance the efficacy of the preventive measures [34, 35]. Control programs for Johne's disease have been established in a number of countries. In Italy, mandatory plans have not been performed in ovine population unlike other nations. Knowledge of the current herd- and sheep-level prevalence is of value today, but it will be more important as a baseline upon which evaluation of control programs can take place.

The highland provinces of Macerata and Ancona in the Marche region (central of Italy) have a sizeable number of dairy ovine populations that produces significant quantity of milk for cheese production and lambs for meat per annum. The farmers in this region have shown increased anxiety to know about the *MAP* prevalence of their herds especially those that ones involved in cheese production using unpasteurised ovine milk and the farmers that observe an unexpected decrease in milk production in their flocks. Very little is known on the epidemiology of ovine paratuberculosis in the central Italy, and *MAP* seroprevalence in the Marche region is unknown.

The objectives of the present study were as follows: (1) to estimate the individual- and herd-level seroprevalence of *MAP* among ovine dairy flocks of Ancona and Macerata provinces of Marche region, Italy and (2) to observe epidemiological factors and to examine their association with *MAP* seroprevalence among adult ovine herds in the two provinces of central Italy.

## 2. Materials and Methods

### 2.1. Study Design

A stratified study was designed in the Marche region of central Italy. The epidemiological unit of concern was the herd.

### 2.2. Target Population and Sample Size

At the beginning of the study, July 2008, the ovine herd consistence in Ancona and Macerata provinces registered in the Italian Anagrafe Nazionale Zootecnica archive [36] (http://statistiche.izs.it/Zootecniche/) is reported in Table 1.

The following formula was used to calculate the sample herd size: *n*_inf _ = (*P*)(1 − *P*)*Z* ^2^/*d* ^2^ [37], where *n*_inf _ = sample size for infinite population; *P* = estimated prevalence of infection [as a decimal: 0.04 (4%)]; *Z* = degree of confidence (*Z* = 1.96 for 95% of confidence); *d* = maximum difference between observed and the true prevalence that we are willing to accept [as a decimal: 0.10 (10%)]. Then, to estimate the required sample size (*n*_fin_) for a finite population (*N*), the following conversion was done: *n*_fin_ = *n*_inf _/1 + (*n*_inf _ − 1)/*N*. Random sampling of ovine herds was performed using the random number generator and considering the herd progressive number list.

### 2.3. Study Population

The data used in this study came from 2086 dairy and mixed, milk and meat, address production sheep randomly selected and reared in 38 farms representative of the herd population in the two provinces of Marche region: eighteen herds in Ancona province and twenty herds in Macerata province.

A stratified random sampling was carried out. The population was divided into subgroups according to geographical area (province), herd consistence, breed, sheep purpose, lactation stage, and clinical signs. A random sample was taken from each of these strata.

The ovine breeds reared were: “Comisana,” “Fabrianese,” “Massese,” “Sarda,” “Sopravvissana,” and crosses. Most of the herds were for milk production, whereas mixed-purpose sheep were predominant in other herds.

The farms consisted of small (≤500 sheep), medium (500< sheep ≥1000), and large (≥1000 sheep) size consistence and were all semi-intensive herds. A systematic sampling was used to take serum samples for this study. The sampling represented the 10% of herds' consistence. The serum samples were collected from individually identified female sheep aged more than two years old.

### 2.4. Survey Design

Farmers were recruited to the study between September 2008 and July 2010. To enlist 38 dairy sheep farms, forty eight owners were contacted by telephone but ten of them did not agree to participate to an interview questionnaire and farm visit for blood sample collection. The epidemiological form was based on three main topics: animal/herd management and facilities, health status, and the sanitary measures adopted during the introduction of new animals in the flock and the formalities of faecal elimination. Each farm had one visit during the study. When seropositive ewes were found, they were submitted to a faecal sampling in a second visit. 

## 3. Laboratory Work

### 3.1. Sampling

During the Annual Official Brucellosis Eradication Campaign, between September 2008 and July 2010, blood samples were collected from 2086 randomly chosen breeding ewes in lactation, aged over two years and reared in 38 farms with or without history of paratuberculosis. At farm visit, the 10% of animals were subjected to a jugular puncture using sterile anticoagulant-free vacutainer tubes. All samples were taken to the Infectious Diseases Laboratory of the School of Veterinary Medical Sciences of the University of Camerino where, after centrifugation, the sera were harvested and stored at −20°C until laboratory analysis was performed. Faecal samples were collected from seropositive sheep in all positive herds for bacteriological culturing and Ziehl Neelsen staining. Diarrhoea and wasting were observed in some sheep during sampling.

### 3.2. Serological Investigations

All blood samples were analysed by a commercial indirect ELISA following the instructions of the manufacturer (ELISA Paratuberculosis kit; Institut Pourquier, France). The specificity of this test was increased by preabsorption with *Mycobacterium phlei *antigen [38]. Wash steps were completed with an automated washer. Double-positive and single-negative control samples were included in all the series of ELISA; in addition to the negative and positive control samples provided by the manufacturer, two internal control serum samples were run on each plate. The cut-off as defined by the manufacturer was Sample to Positive ratio greater than or equals to 0.350 and the ratio between the mean positive control OD and the negative control OD greater than or equal to 3. Briefly, the controls and serum samples were diluted at 1 : 20 and preincubated with *M. phlei* extract which assists in binding unspecific antibodies. After washing, a diluted antiruminant horseradish peroxidase was dispensed per well in order to detect the presence of bounded antibodies. Then the Tetramethylbenzidine (TMB) enzyme substrate was used. The reaction was stopped by 1M HCl solution, and the optical density was measured at 450 nm (OD_450 nm_) using a Multiskan Ascent ELISA reader (Labsystem, Finland). All samples were tested in single and the optical density readings at 450 nm were used for the analyses. The manufacturer recommended 70% as the cut-off for positive samples. The sera within the range of 60–70% were classified as doubtful, while samples below 60% were considered as negative. The generated positive/negative binary outcome was used in all statistical analyses in this study. Doubtful samples were classified as negative and were not included in the statistical analysis of results.

In order to evaluate the true prevalence, the Rogan-Gladen [39] estimator {P = [AP + (SP − 1)]/[SE + (SP − 1)]} was used to convert apparent prevalence values, where P = True prevalence, AP = Apparent prevalence, SP = Test specificity, and SE = Test sensitivity. An estimation had previously been made about the Pourquier's ELISA performances in ovine serum and a sensitivity of 34.9% and a specificity of 98.8% were considered [40].

### 3.3. Bacteriology (Microscopy and Culture)

Ziehl-Neelsen (ZN) stained smears of faeces were examined microscopically and a presumptive diagnosis of paratuberculosis were made when clumps of small strongly acid-fast bacilli were found.

Culture was carried out on individual faecal sample, and a standardized basic method was used as described in the OIE terrestrial manual [5] with some modification.

Briefly, 2-g aliquot was mixed with 35 mL of sterile distilled water and shaken for 30 min. Large debris was allowed to settle for 45 min at room temperature. The aqueous layer was removed and centrifuged at 2500 g for 20 min. The resulting pellet was processed through a decontamination procedure [41]. First, the pellet was resuspended in 0.9% hexadecylpyridinium chloride (HPC; Sigma-Aldrich, Italy) in half strength (0.5×) brain heart infusion broth (BHI; Becton Dickinson), incubated overnight at 37°C, and then centrifuged at 2500 rpm for 20 min to form a pellet. The pellet was resuspended in 1.2 mL 0.5 × BHI containing nalidixic acid (100 *μ*g/mL), vancomycin (100 *μ*g/mL), and amphotericin B (50 *μ*g/mL) (Sigma-Aldrich, Italy) and incubated overnight at 37°C. Herrold's egg yolk medium (HEYM) agar slant containing antibiotics was inoculated with 0.2 mL of the decontaminated inoculum. All cultures were incubated at 37°C for up 20 weeks, with periodic visual assessment. Identification of *MAP* colonies was based on cultural, microscopic, and dependence to mycobactin J for growth. Typical bacterial morphology was confirmed by ZN-staining and by PCR (IS*900*) [42].

## 4. Outcome Variables and Statistical Analysis

A herd was defined as seropositive when one or more sheep in the herd were tested seropositive by serum ELISA test. The herd seroprevalence of *MAP* was calculated from the number of seropositive sheep divided by the total number of sheep tested at visit. Herds were dichotomized above and below the mean herd seroprevalence. Data from questionnaires, serological analysis, ZN staining, faecal culture, and molecular investigations were stored in a database and analysis was performed using a statistical software (STATA version 5; STATA Corporation, College Station, Texas, USA). The serological results were compared by province, herd size, breed and kind of production, lactation stage, and clinical signs.

Descriptive statistics were calculated to determine the apparent *MAP* prevalence at animal and herd levels. Confidence intervals at 95% of the means of clustered samples were calculated as outlined by Thrusfield [43]. Statistical differences from the ELISA results were evaluated by Chi square test. *P* Values less than 0.05 were considered significant.

## 5. Results

The serological investigation was performed on 2.24% of the total sheep reared in the two provinces of the Marche region. Thirty-eight randomly selected farms out of 517 dairy and mixed sheep farms representing 7.35% of the total ovine farms in the areas of study: 7.14% and 7.27% for Ancona and Macerata provinces, respectively, were evaluated. A total of 2086 animals over 2 years of age from the 38 ovine flocks were sampled for serological screening analysis. Mean and median flock sizes were 549 and 400 animals, respectively, and ranged from 110 to 1500 animals. Descriptive statistics of the study population in the two provinces are stated in Table 2. 

The descriptive statistics on herd and sheep level and *MAP *seroprevalence for the dairy and mixed herds are presented in Table 3. 

**Figure 1:**
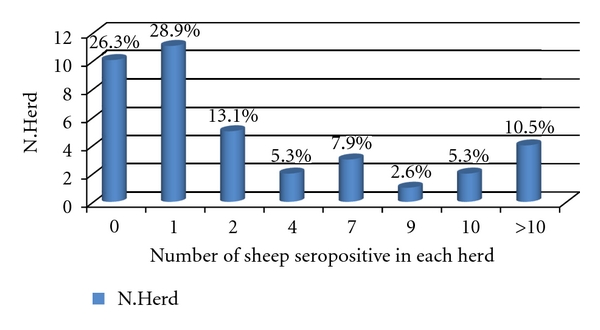
Number and percentage distribution of seropositive sheep over herds.

*MAP* antibodies were detected in 28 farms equal to 73.7% (CI_95_ 59.0–88.3, *n* = 38) of the total herds evaluated. In particular, 11 herds (28.9%) had a single seropositive sheep each and the remaining 17 herds (44.7%) had 2 or more seropositive animals (Figure 1).

In all, a total of 129 seropositive sheep were detected in the two provinces with an apparent prevalence value of 6.29% (CI_95_ 5.23–7.34; *n* = 2086). 

**Figure 2:**
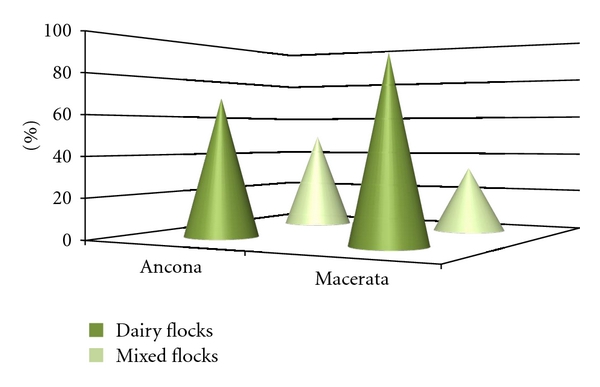
Herd seroprevalence of *MAP* in dairy and mixed flocks of Ancona and Macerata provinces.

**Figure 3:**
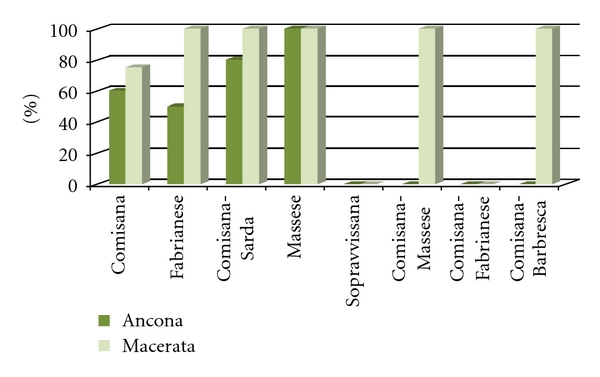
Herd seroprevalence of *MAP* in relation to ovine breeds in Ancona and Macerata provinces.

When analysing *MAP* seroprevalence at the herd-level by province, consistence, herd type, and clinical signs, significant differences were observed between: dairy (88.2%; CI_95_ 71.2–105.3, *n* = 17) and mixed production herds (33.3%; CI_95_ 110.1–176.5, *n* = 3: *χ* ^2^ = 4.80, *P* = 0.0284) in Macerata province (Figure 2), early/late (90.0%; CI_95_ 67.4–112.6) and peak lactation herds (37.5%; CI_95_ 5.8–80.8: *χ* ^2^ = 5.51, *P* = 0.0189) in Ancona province and between the overt disease seropositive and seronegative herds (*P* < 0.05). The herd seroprevalence in relation to breeds is shown in Figure 3.

9 out of 12 herds with small consistence were found positive for* MAP *(75.0%; CI_95_ 46.3–103.7) in both provinces, while different seroprevalence values were recorded for medium herds: 80% (CI_95_ 24.5–135.5, *n* = 5) in Macerata versus 0% (CI_95_ 1.0-1.0, *n* = 3; *χ* ^2^ = 4.80, *P* = 0.0285) in Ancona territory. The frequencies of antibody response against *MAP *infection in the different ovine farms consistence, breeds, and provinces are summarised in Table 4.

**Figure 4:**
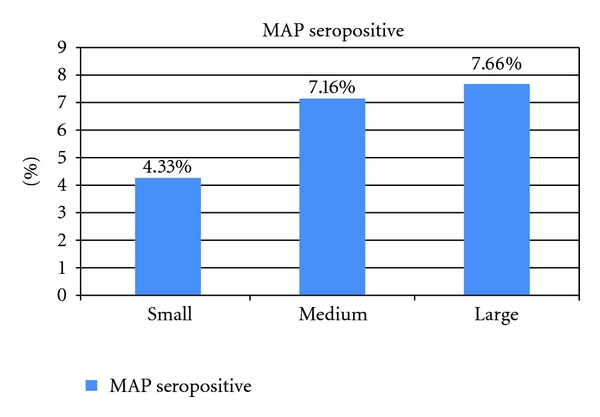
Distribution of *MAP* seropositive sheep in the herd consistence of the studied areas.

A highly significant difference was revealed comparing the seropositivity of *MAP* infection in the large herds and in the small herds (*χ* ^2^ = 4.20, *P* = 0.04) (Figure 4).

The herd seroprevalence in Macerata province was higher in peak (88.9%; CI_95_ 63.3–114.5, *n* = 9) than at early and/or at the end of lactation (72.7%; CI_95_ 41.3–104.1, *n* = 11; *χ* ^2^ = 0.81, *P* = 0.3687). Similar values were observed in Ancona's ovine farms where 9 out of 10 (90.0%; CI_95_ 67.4–112.6) resulted positive at early/late of lactation time, while 3 out 8 (37.5%; CI_95_ −5.8–80.8) at peak lactation. The difference was significant (*χ* ^2^ = 5.51, *P* = 0.0189).

When clinical signs were considered, a significant difference was found in herd seroprevalence. In Macerata province the clinical signs were observed only in seropositive farms (*n* = 16), while 11 out of 12 seropositive herds of Ancona territory (91.7%; CI_95_ 73.3–110.0) reared at least one sheep showed diarrhoea and/or weight loss (*χ* ^2^ = 10.66, *P* = 0.0011).

**Figure 5:**
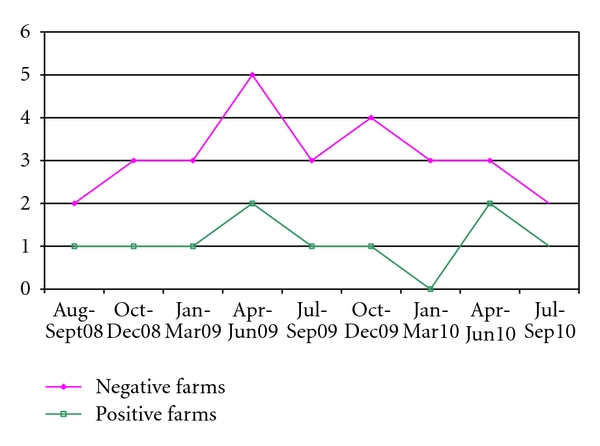
Seasonal distribution of *MAP* seropositive and seronegative farms in the two provinces.

In relation to the period of sampling, an increase of the positive herds is noticed in spring and in autumn but the difference was not significant (*P* = 0.071) (Figure 5). Moreover, by considering two periods in the year, the 68.2% of farms sampling in spring-summer resulted seropositive *versus* the 81.2% of seropositive herds tested in autumn and winter (*χ* ^2^ = 0.82, *P* = 0.3664).

A homogeneous seasonal sampling distribution was revealed in relation to the province and the herd size (Table 5). The Comisana breed is the most represented in the four seasonal samplings. 

Considering the totally examined animals (*n* = 2086), 129 seropositive sheep were found, equal to an overall sheep-level seroprevalence of 6.29% (CI_95_ 5.23–7.34) for the investigated provinces. At the sheep level, significant differences were recorded between *MAP *seropositives in Macerata (7.51%; CI_95_ 5.93–9.08, *n* = 1079) and Ancona province, (4.93%; CI_95_ 3.57–6.30, *n* = 973; *χ* = 5.75, *P* = 0.0165), mixed (1.82%; CI_95_ 0.04–3.60; *n* = 220) and dairy sheep (6.82%; CI_95_ 5.67–7.98, *n* = 1832; *χ* = 8.35, *P* = 0.0039); sheep at early/late lactation (5.16%; CI_95_ 3.86–6.47; *n* = 1104,) and those at peak lactation (7.59%; CI_95_ 5.90–9.28, *n* = 948; *χ* = 5.12, *P* = 0.0237), small (4.33%; CI_95_ 2.88–5.77; *n* = 763) and medium or large farms consistence (*χ* = 7.47, *P* = 0.0063).

*MAP* infection was confirmed in eleven (12.6%, CI_95_ 3.9–21.7; *n* = 87) and in thirteen (14.9%, CI_95_ 4.6–25.2; *n* = 87) faecal samples by bacteriological culture and Ziehl Neelsen staining, respectively. Twenty-three sheep were not detected in the second visit because of selling or death and the remaining nineteen bacteriological cultures resulted contaminated. *MAP* IS900 genome was confirmed by PCR performed on colonies obtained from each of the faecal bacteriological positive sample. 

The true prevalence of *MAP* infection at sheep and herd level in the two provinces was estimated to be 15.1% ± 7.3%. (at 95% confidential limit); 18.7% in Macerata, and 11.1% in Ancona province [39, 40].

## 6. Discussion

Serological investigations have been described as effective tools in the establishment of the prevalence of *MAP* infection in a herd, and also to screen and confirm the diagnosis of paratuberculosis in animals that present compatible clinical symptoms [44]. A comprehensive understanding of *MAP* prevalence, incidence, and epidemiological patterns in ovine flock is of tangible value to facilitate the design of prevention, and control programmes aim at reducing or more preferably eliminate *MAP* from farms. In this light, test selection is of critical importance in the design of such control programmes. The test selected for this current study is quick and relatively easy to perform in contrast to the culturing method which is laborious and requires an incubation period of about 8–16 weeks or more. Moreover, serological analysis would furnish both diagnosis and prognosis of the disease. In this study, an overall sheep-level and herd-level seroprevalence of 6.29% and 73.6% were obtained for the investigated territories.

The apparent prevalence observed in ovine dairy flocks sampled in this study (6.29%) supports the reports of previous researches carried out in cattle [6, 45] whereby the seroprevalence of *MAP* was observed to increase with age of animals and in relation to the herd size. Environmental factors and density-dependent effects can help in *MAP* rampant dispersal.

In this study, herd size was strongly associated with a seropositive herd status. In the large herds a higher percentage of sheep were over 2 years of age than those ones reared in the smaller herds. Thus are more likely to sample and test positive ewes due to a higher adult antibody production or because of herd size or management effects. The within herds variation of *MAP* seroprevalence, observed in the different herds consistence in this study, may be attributed to host variability in antibody production and protein enteropathy in response to *MAP* infection.

The possible within herd transmission dynamic of *MAP* could occur by continuous new *MAP* infection in lambs and high seroprevalence with eventual contamination of the environment or by transmission from dam to lambs and lamb to lamb. The high seroprevalence flocks can serve as permanent reservoirs of *MAP* that may infect other flocks via sheep movements and extensive grazing.

The seroprevalence observed in this study is dissimilar to that reported in sheep population of Umbria region and in Trapani province of Italy where the true and apparent seroprevalences were 4.8% [46] and 3.4% [47], respectively. In Australia, the prevalence was estimated to be in the range of 2.4%–4.4% [48], while in northern Greece, the herd prevalence of *MAP* infection in sheep flocks was estimated to be 21.1% [49]. Seroprevalence study of *MAP* infection in goat dairy flocks in France revealed an apparent and estimated true prevalence of 55.2% and 62.9% at herd level, while at individual animal level they were 2.9% and 6.6%, respectively [50]. However, what obtained in our study is much lower than the high seroprevalence observed in the predominantly sheep and goat flocks in the Madrid region in Spain where an apparent prevalence of 11.7% and estimated true prevalence of 44% were observed [51].

The seroprevalence of *MAP* infection was noted to be higher in Macerata than in Ancona province at both sheep and herd levels, and the difference was quite significant. The herd management practices and the animal introduction checking could be the most likely reasons for the observed difference. Good animal husbandry practice, cleanliness of the farm, manure handling, newborn-lamb care, and restriction of contact between lambs and mature animals were observed in a greater number of farms in Ancona province than in Macerata province during farms' visit [52–54]. Furthermore, a higher percentage of sheep sampled in this study in Ancona province was at early or late stage of lactation while the majority of sheep sampled in Macerata province were at peak stage of lactation. Thus, taken into consideration the postulated hypothesis of a decreased IgG concentration in serum and an increased concentration of IgG in milk at early or late lactation compared with that of peak lactation [55], the stage of lactation could be another possible reason for the difference in *MAP* seroprevalence observed in the two provinces. An in-house comparative study carried out on milk and serum samples postulated a substantial agreement between serum and milk ELISA results (kappa value = 0.67), higher at early and late (*k* = 0.78) than in peak (*k* = 0.54) lactation, in agreement with the Immunoglobulin G (IgG) rising in the milk in the beginning and at the end of lactation (unpublished data).

We advocate strict biosecurity measures within and without the farms for proper containment of the infection. Also, sufficient housing space should be created in the large farm consistence to prevent the animal-to-animal closeness and reduce density-dependent effects which commonly lead to transmission of contagious infections. We also strongly recommended the implementation of a combination of both husbandry changes and test-and-cull methods in *MAP* subclinically infected ovine flocks without overt disease as control strategy for eliminating the pathogen in the ovine farms and preventing their spread to other farms. In *MAP*-infected farms with overt disease, vaccination is advocated for it would prevent more clinical cases, ameliorate the health status of animals exhibiting clinical symptoms, greatly reduce bacterial shedding and thus control contamination risks, and finally may lead to increase production at a highly profitable benefit-to-cost ratio.

## 7. Conclusions

This is the first large-scale study of *MAP *seroprevalence in the ovine population of Marche region in central Italy although until now only two provinces were studied. Serological investigations have been described as effective tools in the establishment of the prevalence of *MAP* infection in a herd, as a useful technique from which animal herd owners could make management decisions [32], and also for confirming the diagnosis of paratuberculosis in animals that present compatible clinical symptoms [56, 57]. The present study can be useful as a confirmatory finding to ascertain the endemicity of JD in the ovine dairy herds in Marche region. The provinces investigated have a rural history with zootechnical farms distributed in the sampled territory homogeneously. In almost all herds, raw milk is used to produce cheese for consumers in own laboratories close to the farm. 

The results are remarkable especially considering the debate on the possibility of *MAP* playing a role in the aetiology of Crohn's disease [8, 14] and human Type I diabetes [15, 16]. The ingestion of contaminated ovine milk and cheese manufactured from raw ovine milk might lead to transmission of *MAP* to humans. Moreover, the Italian normative (Intesa Stato Regioni of 25/1/2007), that defines the criterions of acceptability of raw milk sold directly to consumers, individualizes analytical parameters for the most important bacterial agents of food-borne illnesses but these do not include pathogens like *MAP*. Thus a precautionary approach from public health authorities should be warranted and further investigations are needed to estimate the potential risk for consumers' health.

A comprehensive understanding of *MAP *prevalence, incidence, and epidemiological patterns in ovine flock is of tangible value to facilitate the design of prevention and control programmes aimed at reducing or more preferably eliminate *MAP* from farms in Marche region. 

Knowledge of the current herd- and sheep-level prevalence is of value today, and it is expected that this information will help to prioritize and direct future research and control programs in the region and, further, will be integrated into any national control campaigns.

The clustered distribution of ovine paratuberculosis in Ancona and Macerata provinces of central Italy can be managed and eliminated from sheep flocks with stringent management combined with frequent testing and culling, or by vaccination combined with management of faecal-oral transmission. The frequency of testing and level of management intervention should be determined by each farm's abilities, priorities, and finances. Furthermore, efforts should be made through the appropriate government institutions and through sheep breeder associations to recognize flocks with no history of infection or at low risk of being infected using standardized national certification guidelines. The test negative farms will serve as important source to obtain sheep for the establishment of low risk flocks. 

In addition, the importance of biosecurity measures should be emphasized to avoid a flock becoming infected through the purchase of a sheep. Pilot voluntary control programs have been developed in dairy cattle farm aiming to gradually decrease the prevalence of disease in participating herds. In ovine population, the program could start with a risk assessment evaluation, and then with an implementation of management strategies to prevent transmission of infection, by testing animals before parturition. Based on likelihood ratio approach, the farm should be classified in different categories. Lamb feeding with colostrum and milk should be allowed only from negative ewes: the doubtful and weak negative sheep retested after parturition by molecular techniques. The serum is best suited as the most reliable, fastest, and easiest means of screening ovine flock for the detection of *MAP* infection, but further studies are necessary to determine if the ELISA for individual and bulk tank ovine milk samples can create opportunities for a cheaper and more feasible testing scheme in ovine Johne's disease diagnosis.

## Figures and Tables

**Table 1:** Number of herd consistence recorded by Italian Anagrafe Nazionale Zootecnica archive (National Livestock Population) (http://statistiche.izs.it/Zootecniche) on 31st July 2008.

	Number of herds	
	Ancona province	Macerata province
Milk sheep	29	35
Mixed sheep	213	240

Total	**242**	**275**

**Table 2:** The sheep population in the evaluated herds in Ancona and Macerata provinces.

Study population	Variables	Number of herds		
		Macerata	Ancona	N. Total/Percentage
	Mean	550 (min: 110; max: 1500)	548 (min: 130; max: 1500)	549 (min: 110; max: 1500)
	Median	380	490	400
				
Herd type	Dairy	17	16	33 (86.8%)
	Mixed production	3	2	5 (13.2%)
				
	Comisana	8	10	18 (47.4%)
	Comisana-Sarda	6	5	11 (28.9%)
	Fabrianese	1	2	3 (7.9%)
Breed	Massese	1	1	2 (5.3%)
	Sopravvisana	1	—	1 (2.6%)
	Comisana-Massese	1	—	1 (2.6%)
	Comisana-Fabrianese	1	—	1 (2.6%)
	Comisana-Barbaresca	1	—	1 (2.6%)
				
	Spring	6	7	12 (31.6%)
	Summer	5	5	10 (26.3%)
Season of sampling	Autumn	5	4	9 (23.7%)
	Winter	5	2	7 (18.4%)
				
Lactation stage	Early/late	11	10	21 (55.3%)
	Peak	9	8	17 (44.7%)
				
	Small (≤500)	12	12	24 (63.1%)
Herd consistence	Medium (>500 ≤1000)	5	3	8 (21.1%)
	Large (≥1000)	3	3	6 (15.8%)
				
Clinical signs	Present (diarrhoea and/or wasting)	16	11	27 (71.0%)
	Absent	4	7	11 (28.9%)

**Table 3:** Descriptive statistics for dairy and mixed sheep herds.

	Total herds	Dairy	Mixed
Number of herds	38	33	5
Mean number sheep sampled/herd	55	57	38
Number of seropositive sheep/herd	3.6	4.1	0.8
Number of seropositive sheep/positive herds	5.1	4.1	1.5
% Herds ≥1 seropositive sheep	73.7	78.8	40.0

**Table 4:** Herd seroprevalence in relation to the breed and herd consistence in Macerata and Ancona provinces.

	% Herd seroprevalence (CI_95_)							
			Herd consistence					
Breed	Total herds		Small (≤500 sheep)		Medium (500 < *n* ≥ 1000)		Large (≥1000 sheep)	
	MC	AN	MC	AN	MC	AN	MC	AN
*Comisana *	75.0 (36.3–116.7) *n* = 8	60.0 (23.1–96.9) *n* = 10	60.0 *n* = 5	66.7 *n* = 6	100 *n* = 1	0 *n* = 2	100 *n* = 2	100 *n* = 2

*Fabrianese *	100 (1-1) *n* = 1	50.0 (−585.3–685.3) *n* = 2	100 *n* = 1	50.0 *n* = 2	—	—	—	—

*Comisana-Sarda*	100 (1-1) *n* = 6	80.0 (24.5–135.5) *n* = 5	100 *n* = 4	100 *n* = 4	100 *n* = 2	0 *n* = 1	—	—

*Massese *	100 (1-1) *n* = 1	100 (1-1) *n* = 1	—	—	—	—	100 *n* = 1	100 *n* = 1

*Sopravissana *	0 *n* = 1	—	—	—	0 *n* = 1	—	—	—

*Comisana*-*Massese *	100 (1-1) *n* = 1	—	100 *n* = 1	—	—	—	—	—

*Comisana*-*Fabrianese *	0 *n* = 1	—	0 (*n* = 1)	—	—	—	—	—

*Comisana*-*Barbaresca *	100 (1-1) *n* = 1	—	—	—	100 *n* = 1	—	—	—

MC = Macerata province; AN = Ancona province.

**Table 5:** Herd size seasonal sampling.

Season	N. Herd size		
	Small	Medium	Large
Spring	8	2	2
Summer	7	2	1
Autumn	5	2	2
Winter	4	2	1
